# Trends in prevalence of guideline‐based use of lipid‐lowering therapy in a large health system

**DOI:** 10.1002/clc.23347

**Published:** 2020-02-27

**Authors:** Abbey C. Sidebottom, Marc C. Vacquier, Joseph C. Jensen, Steven M. Bradley, Thomas Knickelbine, Craig Strauss, Michael D. Miedema

**Affiliations:** ^1^ Care Delivery Research, Allina Health Minneapolis Minnesota; ^2^ Minneapolis Heart Institute Foundation Minneapolis Minnesota; ^3^ Minneapolis Heart Institute Minneapolis Minnesota

**Keywords:** cholesterol, guidelines, population health, statin

## Abstract

**Background:**

The 2013 ACC/AHA (American College of Cardiology/American Heart Association) cholesterol guidelines provided an evidence‐based rationale for the allocation of lipid‐lowering therapy based on risk for atherosclerotic cardiovascular disease (ASCVD). Adoption of these guidelines was initially suboptimal but whether this has improved over time remains unclear.

**Hypothesis:**

Prevalence of guideline‐based statin therapy will increase over time.

**Methods:**

Electronic health record data were used to create two cross‐sectional data sets of patients (age 40‐75) served in 2013 and 2017 by a large health system. Data sets included demographics, clinical risk factors, lipid values, diagnostic codes, and active medication orders during each period. Prevalence of indications for statin therapy according to the ACC/AHA guidelines and statin prescriptions were compared between each time period.

**Results:**

In 2013, of the 219 376 adults, 57.7% of patients met statin eligibility criteria, of which 61.3% were prescribed any statin and 19.0% a high intensity statin. Among those eligible, statin use was highest in those with established ASCVD (83.9%) and lowest in those with elevated ASCVD risk >7.5% (39.3%). In 2017, of the 256 074 adults, 62.3% were statin eligible, of which 62.3% were prescribed a statin and 24.3% a high intensity statin. In 2017, 66.4% of statin eligible men were prescribed a statin compared to 57.4% of statin eligible women (*P* < 0.001). The use of ezetimibe (3.6% in 2013, 2.4% in 2017) and protein convertase subtilisin/kexin type 9 inhibitors (<0.1% and 0.1%) was infrequent.

**Conclusion:**

In a large health system, guideline‐based statin use has remained suboptimal. Improved strategies are needed to increase statin utilization in appropriate patients.

## 1 INTRODUCTION

In 2013, the American College of Cardiology (ACC) and American Heart Association (AHA) released guidelines for the treatment of blood cholesterol, recommending that statin therapy be allocated to those at elevated risk of atherosclerotic cardiovascular disease (ASCVD), including patients with established ASCVD, diabetes, or a low‐density lipoprotein cholesterol (LDL‐C) ≥190 mg/dL, as well as patients with an estimated 10‐year ASCVD risk of ≥7.5%.^1^ This risk‐based approach was a significant shift from the prior adult treatment panel III guidelines,^2^ and was reinforced in the recent 2018 ACC/AHA cholesterol guideline update.^3^

Subsequent analyses suggested that implementation of the 2013 guidelines would lead to a dramatic increase in statin use across the United States, with US National Health and Nutrition Examination Survey (NHANES) data suggesting that almost of half of US adults aged 40 to 75 years are statin eligible by the 2013 guidelines.^4^ The majority of the increase in statin eligibility was seen in older individuals without established ASCVD.^4^, ^5^ However, a subsequent analysis suggested that in the years immediately following publication of the 2013 guidelines, statin utilization minimally changed.^6^

Whether or not compliance with the 2013 ACC/AHA cholesterol guidelines has improved in more recent years is unclear. Statin therapy can significantly reduce ASCVD risk, including in individuals at relatively low ASCVD risk,^7^ and, in contrast to public perception,^8^ statins have an excellent safety profile with minimal side effects in blinded trials.^9^, ^10^ Improved adoption of the 2013 cholesterol guidelines holds the potential to significantly reduce ASCVD rates at a population level.^11^ We therefore aimed to analyze the prevalence of statin eligibility and subsequent rates of statin use in a large Midwestern health system.

## 2 METHODS

### 2.1 Setting and patients

This retrospective cohort study analyzed data from Allina Health, a large Midwestern health system operating 12 hospitals and over 100 clinics (primary care and specialty clinics within Minnesota and western Wisconsin). Data extracts were generated from Allina's electronic health record (EHR). All hospitals and clinics in the Allina Health system use the EpicCare EHR system (Epic Systems Corporation) which Allina has branded Excellian. This study was approved by the Allina Health Institutional Review Board.

**Figure 1:**
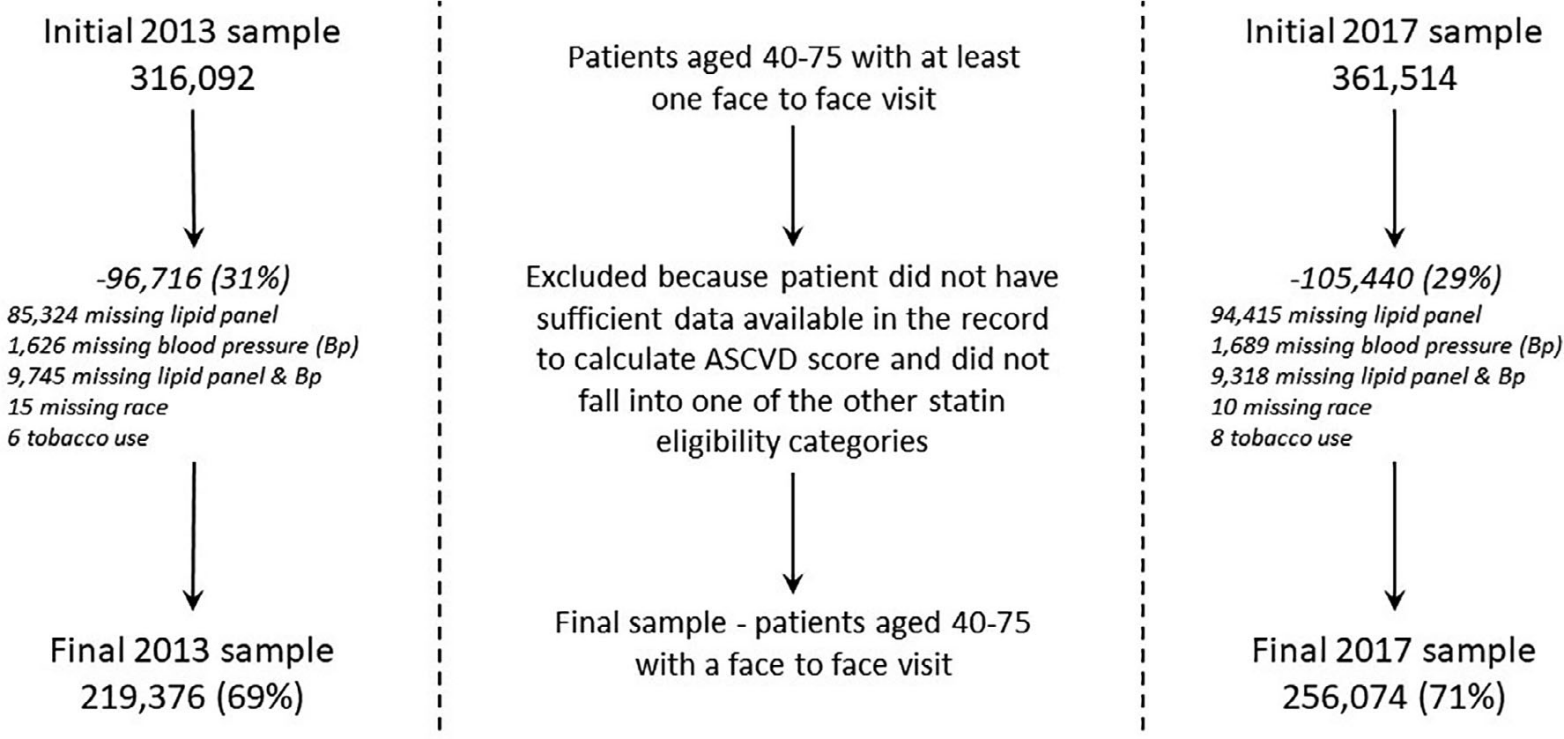
Study sample selection process for 2013 and 2017 data sets. ASCVD, atherosclerotic cardiovascular disease

Two cross‐sectional data sets were created, the first including patients who received care in 2013 and the second, 4 years after the 2013 guidelines were released (2017). We included adults aged 40 to 75 years at the start of each extract time period (ie, 2013 and 2017) with at least one in‐person ambulatory clinic visit during that year including primary care office visits, OB/GYN (obstetrics or gynecology) encounters for patients who were not pregnant, pre‐operation visits, and nurse‐only visits. Pregnant patients were excluded. Individuals were excluded who had opted out of use of their data for research purposes through the Minnesota Research Authorization process (typically <5% of the patient population). After applying the initial inclusion criteria, we then excluded patients with insufficient data to determine statin eligibility. The initial inclusion criteria identified 316 092 patients in 2013 and 361 514 in 2017. Approximately 30% of each of these cohorts was excluded due to lack of sufficient data to determine statin eligibility, leaving 219 376 patients in 2013 and 256 074 patients in 2017 (Figure 1).

Patients excluded due to missing data differed from those who were included in the final sample on several characteristics. In the 2013 cohort, the excluded patients were an average of 4 years younger than the included, and had lower prevalence of hypertension (36% in the excluded vs 55% in the included), and <1% had documented diabetes or heart disease compared to 18% and 14% for these conditions, respectively in the included sample. Differences were very similar for the included and excluded in the 2017 cohort. Additionally, statin use was much lower in those excluded, with 9% of those excluded from the 2013 sample and 10% of those excluded from the 2017 sample having an active order for statins (compared to 43% of those included in the 2013 sample and 44% in the 2017 sample).

### 2.2 Assessment of statin use and eligibility

The use of lipid‐lowering medications was defined by any medication order (indicating a prescription) within a lipid lowering medication category in the EHR from a non‐hospital visit with a start date prior to the end of the study period and no end date prior to the start of the study period. This broad definition capturing “any active order” during the study period was used for the primary analysis. As a sensitivity analysis, we also created a secondary measure that looked at active orders only at the time of the last visit during the study period. Medication orders for a lipid lowering medication were divided into two broad categories: statins and other lipid‐lowering medications. The specific statin and dose was documented for each participant. Rosuvastatin (dose 20‐40 mg/d), atorvastatin (dose 40‐80 mg/d), and simvastatin (dose 80 mg/d) were considered to be high intensity statins. Other lipid‐lowering medications were categorized into three groups: protein convertase subtilisin/kexin type 9 (PCSK9) inhibitors, ezetimibe, and other lipid‐lowering medications which included niacin (vitamin B3), bile sequestrants, fibrates, ethyl esters, and resins. If a patient was taking a medication that combined two different drug categories, they were coded as taking each type of drug included.

The 2013 ACC/AHA cholesterol guidelines define four major indications for statin therapy: (a) a diagnosis of clinical CVD (acute coronary syndromes, history of myocardial infarction, stable angina, coronary or other arterial revascularization, stroke, transient ischemic attack, or peripheral arterial disease of atherosclerotic origin); (b) a diagnosis of diabetes; (c) LDL‐C >190 mg/dL; and (d) an estimated 10‐year CVD risk ≥7.5% (with an LDL‐C 70‐189 mg/dL) according to the Pooled Cohort Equations calculator, which was created in conjunction with the guidelines.^1^ For individuals meeting one of these four criteria, a moderate to high intensity statin is a Class I recommendation for individuals age 40 to 75 years. For individuals with diabetes or a 10‐year CVD risk >7.5%, a risk‐based discussion is recommended prior to consideration of statin therapy. For individuals with a 10‐year CVD risk 5% to 7.5%, a risk‐based discussion is recommended and a moderate intensity statin can be considered (Class IIa recommendation). Individuals with a 10‐year CVD risk <5% are recommended to not undergo pharmacologic treatment to lower their cholesterol.

Patients were classified into statin eligibility categories in a stepwise fashion, first selecting individuals with a diagnosis of CVD, then individuals without known CVD but with a diagnosis of diabetes, then individuals without CVD or diabetes but with an LDL >190 mg/dL, and finally, individuals not meeting the three previous criteria but with a 10‐year CVD risk ≥7.5% according to the Pooled Cohort Equations calculator.^1^ For individuals with an identified race other than white or African American (ie, Asian/Pacific Islander, American Indian), the model developed for white populations was applied. The remaining individuals were stratified into two categories based on their 10‐year CVD risk (5%‐7.5% and <5%).

As a sensitivity analysis, we reanalyzed statin eligibility using estimated off treatment LDL‐C levels for individuals on statin therapy. We assumed a 30% increase in LDL‐C for individuals on a low or moderate intensity statin and a 50% increase in LDL‐C for individuals on a high intensity statin. Individuals were then reclassified into eligibility categories using the same stepwise fashion described above.

### 2.3 Other clinical variables

Patient demographics include age (calculated at of the end of each extract period), sex, race, ethnicity, marital status, preferred language, and insurance type (categorized as Medicaid/ Medicare or private/military). Smoking status, blood pressure, height and weight were collected as the last value available during the extract year. Diagnosis of chronic conditions (diabetes, chronic kidney disease, coronary heart disease) was identified based on the presence of specified ICD values in any visit diagnosis coding prior to the end of the extract period. Highest LDL‐C within 3 years prior to the extract were collected to help capture those with LDL‐C ≥190 mg/dL. Most recent total cholesterol and high‐density lipoprotein cholesterol values were used from the prior 3 years for use in the pooled cohort equation. All active orders (from clinic appointments) for hypertension medication during the extract period were collected. Participants were classified as hypertensive if their last blood pressure value was >140/90 mmHg or if they had an active order for blood pressure medication.

### 2.4 Statistical analysis

Patient characteristics of the final sample were compared to those who were excluded due to missing data (primarily missing lipid panel) for each time period using chi‐square for categorical variables and *t* tests for continuous variables. The prevalence of use of statin and other lipid lowering medications were stratified according to the ACC/AHA guidelines categories. We then performed similar analyses stratified by gender and by race/ethnicity. The data were analyzed using Stata version 15.1 (StataCorp).

## 3 RESULTS

The baseline characteristics of the 219 376 patients in 2013 and the 256 074 patients in 2017 included in the analysis are shown in Table 1. The cohort in 2017 was slightly older with a higher prevalence of coronary heart disease, kidney disease, and diabetes.

**Table 1:** Comparison of 2013 and 2017 study samples

	2013 (n = 219 376)	2017 (n = 256 074)	*P*‐value
Age, mean (SD)	57.4 (9.4)	58.5 (9.5)	<.001
Gender (%)			.775
Male	46.7	46.7	
Female	53.3	53.3	
Race (%)			<.001
African American	3.1	3.6	
American Indian	0.4	0.4	
Asian	2.0	2.6	
Hawaiian/Pacific Islander	0.1	0.2	
White	92.5	91.1	
Multiple	0.3	0.3	
Missing	1.7	1.9	
Hispanic (%)	1.6	2.0	<.001
Preferred language (%)			<.001
English	98.2	97.6	
Spanish	0.4	0.6	
Somali	0.2	0.2	
Other	1.3	1.6	
Marital status (%)			.377
Married	69.9	70.0	
Single	30.1	30.0	
Insurance status (%)			.127
Medicaid/Medicare	23.2	23.0	
Private or military insurance	76.8	77.0	
Diabetes (%)	18.2	19.3	<.001
Chronic kidney disease (%)	3.7	5.1	<.001
Coronary artery disease (%)	13.7	14.9	<.001
Current smoker (%)	12.0	11.5	<.001
Body mass index categories (%)			<.001
Healthy weight (<25)	25.3	24.4	
Overweight (25‐29)	33.6	33.3	
Obese (30+)	38.1	40.4	
Missing	3.0	1.9	
Body mass index, mean (SD)	29.4 (6.49)	29.6 (6.66)	<.001
Blood pressure, mean (SD)			
Systolic BP (mmHg)	122.3 (14.6)	123.9 (15.2)	<.001
Diastolic BP (mmHg)	74.4 (9.4)	75.3 (9.6)	<.001
Hypertension (%)	55.3	58.0	<.001
Blood pressure data available (%)	99.4	99.5	<.001
Lipid data available (%)	95.3	94.0	<.001

In 2013, 57.7% of patients met statin eligibility criteria, with the highest prevalence of statin eligibility due to ASCVD risk ≥7.5% (25%), followed by 16.5% with a clinical ASCVD diagnosis, 13.1% with diabetes, and 3.1% with an LDL‐C ≥190 (Table 2). Of those eligible for a statin, 61.3% were prescribed a statin with 19.0% on a high intensity statin. Among statin eligible patients, the highest prevalence of statin use was in those with established ASCVD (83.9%) while the lowest prevalence of use was in those with an estimated 10‐year ASCVD risk ≥7.5% (39.3%). In 2013, use of ezetimibe (3.6%) was infrequent and very few patients were prescribed a PCSK9 inhibitor (<0.1%). In 2013, 2.4% of the sample was listed as having a statin allergy. However, 78% of those with a statin allergy had an active order for statin therapy, indicating they were tolerating a different dose or a different statin.

**Table 2:** Prevalence of statin and other lipid lowering medication prescriptions stratified by ACC/AHA cholesterol guideline eligibility criteria, for patients age 40 to 75 in 2013 and 2017

	Clinical ASCVD	LDL‐C (≥190)	Diabetes	ASCVD risk (≥7.5%)	Total statin eligible	ASCVD risk (5%‐7.5%)	ASCVD risk (<5%)
2013 Cohort (n = 219 376)	36 166 (16.5)	6833 (3.1)	28 685 (13.1)	54 797 (25.0)	126 481 (57.7)	19 734 (9.0)	73 161 (33.3)
Any statin (%)	83.9	68.0	73.1	39.3	61.3	30.3	14.9
High intensity statin (%)	38.4	20.7	18.4	6.3	19.0	4.8	2.1
Other lipid lowering medications							
PCSK9 inhibitors (%)	0.0	0.0	0.0	0.0	0.0	0.0	0.0
Ezetimibe (%)	7.2	3.9	3.5	1.2	3.6	0.8	0.4
Other^a^ (%)	12.0	8.1	12.0	5.6	9.6	4.1	2.3
No statins or other LLM (%)	14.0	29.3	23.9	57.8	36.1	67.5	83.7
2017 Cohort (n = 256 074)	46 799 (18.3)	8807 (3.4)	34 965 (13.7)	68 996 (26.9)	159 567 (62.3)	21 868 (8.5)	74 639 (29.1)
Any statin (%)	83.7	59.4	75.2	41.6	62.3	26.1	11.5
High intensity statin (%)	49.9	20.9	22.3	8.5	24.3	5.5	2.3
Other lipid lowering medications							
PCSK9 inhibitors (%)	0.2	0.7	0.0	0.0	0.1	0.0	0.0
Ezetimibe (%)	5.2	2.5	1.9	0.8	2.4	0.4	0.2
Other^a^ (%)	7.6	4.0	8.4	3.5	5.8	2.6	1.4
No statins or other LLM (%)	15.1	38.6	23.1	56.8	36.2	72.6	87.5

Abbreviations: ACC/AHA, American College of Cardiology/American Heart Association; ASCVD, atherosclerotic cardiovascular disease; LDL‐C, low‐density lipoprotein cholesterol; LLM, lipid lowering medication; PCSK9, proprotein convertase subtilisin/kexin type 9.

a Other lipid lowering medications includes niacin, fibrates, ethyl esters, resins, bile sequestrants.

The prevalence of statin eligibility was slightly higher in 2017 (62.3% overall) with a similar distribution of patients across the eligibility categories compared to 2013 (Table 2). In 2017, there was minimal change in use of statin therapy compared to 2013 with 62.3% of patients prescribed a statin. There was an increase in use of high intensity statins with 24.3% of statin eligible patients on a high intensity statin including 49.9% of those with clinical ASCVD. There was a decrease in use of statin therapy in patients at low ASCVD risk with 11.5% of low risk patient prescribed a statin in 2017 compared to 14.9% in 2013. In 2017, the use of ezetimibe remained infrequent (2.4%) with rare use of PCSK9 inhibitors (0.1%). In 2017, 2.8% of the sample was listed as having a statin allergy but 76% of those with a statin allergy had an active order for statin therapy. Using a more conservative measure for statin use by requiring an active statin prescription during the last documented patient encounter for each year resulted in a slightly lower prevalence of statin use in eligible patients (58.1% in 2013, and 59.5% in 2017).

**Figure 2:**
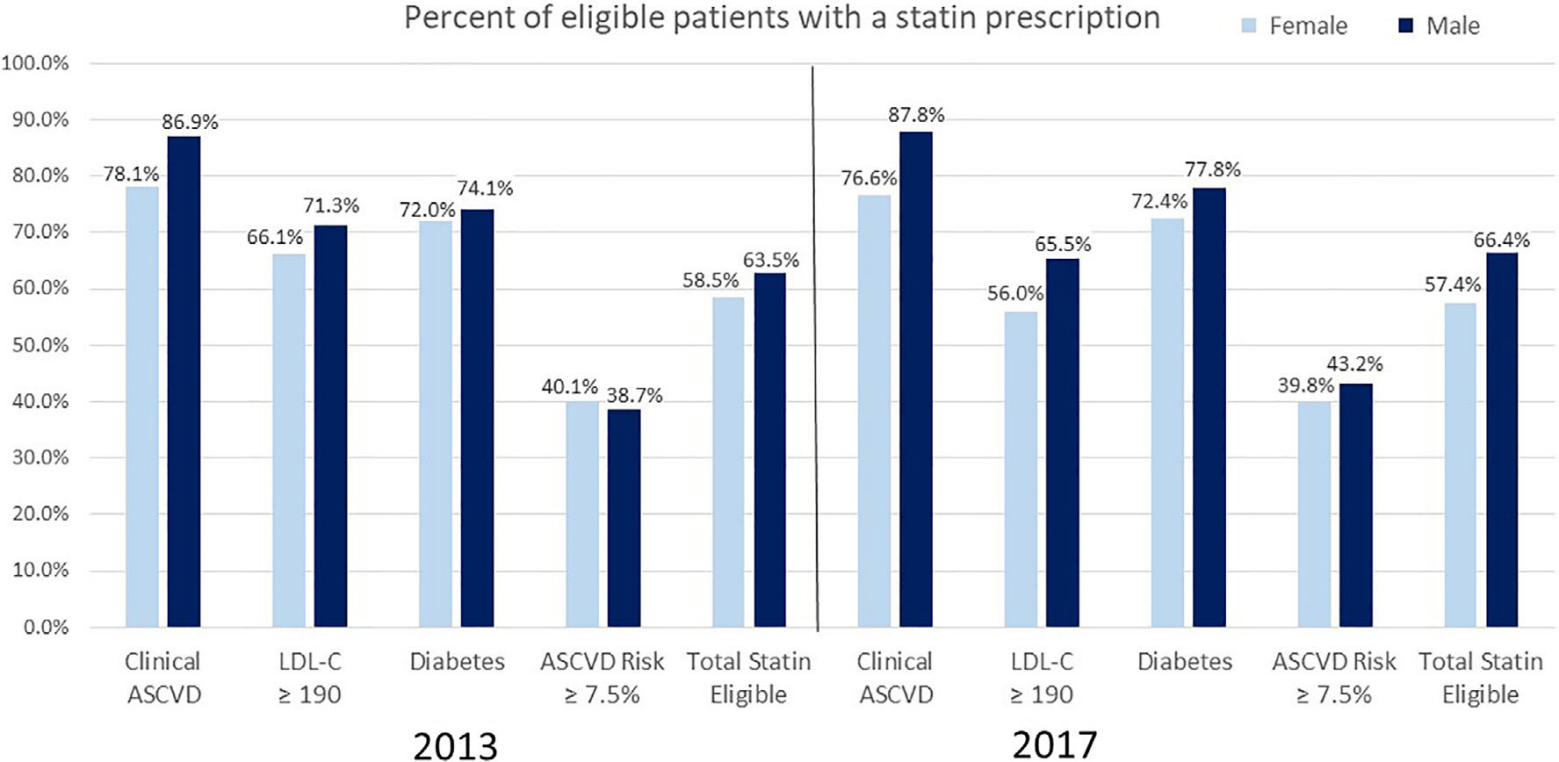
Statin use prevalence by ACC/AHA eligibility categories in 2013 and 2017, by sex. ACC/AHA, American College of Cardiology/American Heart Association; ASCVD, atherosclerotic cardiovascular disease; LDL‐C, low‐density lipoprotein cholesterol

We also examined the use of statin therapy in those statin eligible according to sex. Women were less likely to receive statin therapy in both 2013 and 2017 (Figure 2). In 2017, 72.8% of men were statin eligible, compared to 53.1% of women. Of statin eligible men, 66.4% were prescribed a statin compared to 57.4% of statin eligible women (*P* < .001). Women were less likely to receive treatment within each of the statin eligibility categories (Table 3).

**Table 3:** Prevalence of statin and other lipid lowering medication prescriptions by ACC/AHA cholesterol guideline eligibility criteria for patients age 40 to 75 in 2017, stratified by sex

	Clinical ASCVD	LDL‐C (≥190)	Diabetes	ASCVD risk (≥7.5%)	Total statin eligible	ASCVD risk (5%‐7.5%)	ASCVD risk (<5%)
Female (n = 136 542)	17 006 (12.5)	5680 (4.2)	17 141 (12.6)	32 680 (23.9)	72 507 (53.1)	11 810 (8.7)	52 225 (38.3)
Any statin (%)	76.6	56.0	72.4	39.8	57.4	22.5	8.7
High intensity statin (%)	38.8	18.1	20.4	6.6	18.3	3.9	1.4
Other lipid lowering medications							
PSCK9 inhibitors (%)	0.2	0.5	0.0	0.0	0.0	0.0	0.0
Ezetimibe (%)	4.3	2.3	2.0	0.9	2.1	0.4	0.1
Other^a^ (%)	5.9	3.9	6.8	2.8	4.5	2.0	1.0
No statins or other LLM (%)	22.0	41.8	25.9	58.7	41.0	76.3	90.5
Male (n = 119 532)	29 793 (24.9)	3127 (2.6)	17 824 (14.9)	36 316 (30.4)	87 060 (72.8)	10 058 (8.4)	22 414 (18.8)
Any statin (%)	87.8	65.5	77.8	43.2	66.4	30.3	18.0
High intensity statin (%)	56.2	26.1	24.2	10.2	29.4	7.3	4.3
Other lipid lowering medications							
PSCK9 inhibitors (%)	0.2	0.1	0.0	0.0	0.1	0.0	0.0
Ezetimibe (%)	5.8	2.7	1.8	0.8	2.8	0.5	0.4
Other^a^ (%)	8.6	4.0	9.9	4.2	6.8	3.3	2.5
No statins or other LLM (%)	11.1	32.8	20.4	55.0	32.1	68.2	80.5

Abbreviations: ACC/AHA, American College of Cardiology/American Heart Association; ASCVD, atherosclerotic cardiovascular disease; LDL‐C, low‐density lipoprotein cholesterol; LLM, lipid lowering medication; PCSK9, proprotein convertase subtilisin/kexin type 9.

a Other lipid lowering medications includes niacin, fibrates, ethyl esters, resins, bile sequestrants.

A sensitivity analysis categorizing statin eligibility using estimated off‐treatment LDL‐C levels for those on statin therapy, assuming a 30% and 50% increase in LDL‐C for a moderate and high intensity statins respectively, resulted in a modest shift across eligibility categories with an additional 2% of individuals categorized as LDL‐C >190 mg/dL (5.1% in the sensitivity analysis compared to 3.1% in the original analysis in the 2013 cohort). Additionally, <1% of those classified as borderline or low ASCVD risk in the original analysis were reclassified at ≥7.5% ASCVD risk based on their estimated off‐treatment LDL‐C in the 2013 cohort (25.8% in the sensitivity analysis compared to 25.0% in the original cohort). The modest shift was similar for the 2017 cohort (results not shown).

## 4 DISCUSSION

In an analysis of approximately one‐quarter of a million adults from a large Midwestern healthcare system, we found that the prevalence of statin use in adults eligible by the 2013 ACC/AHA guidelines has minimally changed over time with approximately 60% of statin eligible adults prescribed a statin in 2013 and 2017. There was a modest increase in use of high intensity statins in 2017 compared to 2013 but still only half of patients with clinical ASCVD in 2017 were on a high intensity statin. For adults in 2017 with an estimated ASCVD risk >7.5% eligible for a risk‐based discussion to consider statin therapy, only 42% were on a statin. Additionally, use of ezetimibe and the PCSK9 inhibitors remained infrequent. Further strategies are needed to improve compliance with the ACC/AHA cholesterol guidelines.

### 4.1 The prevalence of statin eligibility in the United States

NHANES data suggested that application of the 2013 ACC/AHA cholesterol guidelines would lead to statin eligibility in 48.6% of the estimated 115.4 million US adults aged 40 to 75 years, ~30% increase in statin eligibility compared to the prior ATP III guidelines.^4^ The majority of those newly eligible were adults over age 60 without established ASCVD. An additional analysis from the atherosclerosis in communities (ARIC) study confirmed the significant increase in statin eligibility in older populations, with near universal eligibility for adults aged 65 to 75 years.^12^ In contrast to the NHANES data, we found a higher prevalence of statin eligibility with approximately 60% of our sample statin eligible in 2013 and 2017. The higher prevalence of statin eligibility is likely due to our sample being limited to individuals who have sought healthcare.

The goal of the 2013 ACC/AHA guidelines was to allocate statins to those most likely to benefit and subsequent analyses have suggested success in this goal. Compared to the 30% increase in eligibility in the general population suggested by the NHANES data, an analysis of over 1000 patients experiencing ST‐elevation myocardial infarction (STEMI) found that pre‐STEMI statin eligibility increased over 100% compared to the ATP III guidelines with 39% of pre‐STEMI patients statin eligible by ATP III compared with 79% statin eligible with application of the ACC/AHA guidelines.^13^

### 4.2 The prevalence of statin use in patients who are statin eligible

Prior data from Pokharel et al analyzed over one million patients from 161 US cardiology practices found that in the adoption of the 2013 guidelines was initially quite modest with 62.1% on a moderate to high intensity statin during the September 2012 to November 2013 time period leading up to the release of the guidelines while 66.6% of patients were on a moderate to high intensity statin during the February 2014 to April 2015 time period after the release of the guidelines.^6^ Similar to our data, this prior study found that use of statin therapy was highest in those with established ASCVD (62.7% and 67% during the two time periods) and lowest in those with estimated ASCVD risk ≥7.5% (41.9% and 46.9% during the two time periods). Our study demonstrated a similar ~60% rate of statin use in statin eligible patients and while the data from Pokharel et al demonstrates that initial adoption of the 2013 guidelines was modest, our data further extends this finding as our second time period was 4 years after the release of guidelines, presumably a more than adequate amount of time to allow adequate dissemination of the guidelines.

Additionally, we found that women were less likely to be prescribed a statin compared to men with almost a 10% absolute lower rate of statin use (57.4% vs 66.4%), a finding that has been seen in previous studies assessing guideline‐based statin use.^14^, ^15^ In our sample, the disparity in statin use between men and women was highest for patients eligible due to clinical ASCVD as opposed to the other three eligibility categories, suggesting that the disparity is higher for secondary prevention than primary prevention. Prior research has demonstrated that women are less likely to receive statin therapy even after adjusting for baseline demographics and other clinical variables including the presence of ASCVD,^15^ further confirming that the gender disparities seen in the treatment of CVD are complex, poorly understood, and in need for further studies.

### 4.3 Reasons for lack of appropriate statin use

There are likely multiple reasons for the modest adherence to guideline‐based statin use. Patients are understandably concerned about statin side effects and observational studies have reported that up to 25% of individuals experience statin‐related side effects.^9^ In our analysis, <3% of individuals had a statin allergy listed and the majority of those individuals had an active order for a different dose or different statin. Prior data from the Patient and Provider Assessment of Lipid Management (PALM) registry reported that fear of side effects was the most common reason for declining statin therapy.^8^ However, the rate of side effects in the blinded statin trials has consistently been similar between the statin and placebo groups. The Anglo‐Scandinavian Cardiac Outcomes Trial—Lipid‐Lowering Arm tested atorvastatin for primary prevention of ASCVD and included a non‐blinded phase after the blinded portion of the trial was completed.^10^ The trial reported no difference in muscle‐related adverse events during the blinded portion of the trial but during the non‐blinded portion of the trial, muscle‐related adverse events in those on statin therapy were reported at a frequency 41% higher (*P* = 0.006) than those not on statin therapy. While these data demonstrate the significant contribution of the nocebo effect to statin intolerance and subsequent lack of appropriate statin use, other factors may be even more important. The aforementioned PALM registry data also demonstrated that approximately 60% of statin eligible adults that were not on statin therapy reported never even being offered a statin by their provider.^8^ Cost may be a potential limiting factor in statin use but nearly all statins are currently generic. From 2013 to 2017, the high intensity statins atorvastatin and rosuvastatin became generic, which is likely a significant driving factor for the increase use of high intensity from 2013 to 2017 seen in our study.

### 4.4 Strengths and limitations

Our analysis has several strengths, including a contemporary analysis of statin utilization, including the specific statin and dosage, in a large sample of men and women from urban, suburban, and rural populations. Use of EHR data allowed identification of ASCVD diagnoses as well as other ASCVD risk factors, allowing for calculation of 10‐year ASCVD risk and placement of patients into appropriate categories of statin eligibility. It also has potential limitations. Statin use is known to vary by geographic location,^16^ making the generalizability of these findings to populations outside the Midwestern United States less reliable. A lack of historical lipid values likely led to misclassification of some individuals with LDL‐C ≥190 mg/dL who were identified and treated prior to our assessment. However, as indicated in our sensitivity analysis, this likely affected 2% of the patient population. Additionally, due to missing data, we were unable to identify statin eligibility for approximately 30% of the patients within this healthcare system and we could only assess statin orders provided within the health system studied.

## 5 CONCLUSION

In conclusion, we found that over one‐third of statin eligible adults in a large Midwestern healthcare system are not currently prescribed a statin, with suboptimal use of high intensity statin therapy, ezetimibe, and PCSK9 inhibitors as well. Despite sound evidence supporting the recommendations in the ACC/AHA cholesterol guidelines, use of statin therapy remains suboptimal and strategies to improve guideline adherence hold the potential to have a significant impact on population rates of ASCVD events.

## CONFLICT OF INTEREST

The authors have no conflicts of interest.
